# Induced pluripotent stem cell-derived endothelial cells promote angiogenesis and accelerate wound closure in a murine excisional wound healing model

**DOI:** 10.1042/BSR20180563

**Published:** 2018-07-31

**Authors:** Zoë E. Clayton, Richard P. Tan, Maria M. Miravet, Katarina Lennartsson, John P. Cooke, Christina A. Bursill, Steven G. Wise, Sanjay Patel

**Affiliations:** 1 Cell Therapeutics and Applied Materials Groups, Heart Research Institute, 7 Eliza Street, Newtown NSW 2042, Australia; 2 Sydney Medical School, University of Sydney, Camperdown NSW 2050, Australia; 3 Faculty of Medicine and Health Sciences, Linköping University, Linköping 581 83, Sweden; 4 Department of Cardiovascular Sciences, Houston Methodist Research Institute, 6670 Bertner Ave, Houston, TX 77030, U.S.A.

**Keywords:** angiogenesis, endothelial cells, induced pluripotent stem cells, wound healing

## Abstract

Chronic wounds are a major complication in patients with cardiovascular diseases. Cell therapies have shown potential to stimulate wound healing, but clinical trials using adult stem cells have been tempered by limited numbers of cells and invasive procurement procedures. Induced pluripotent stem cells (iPSCs) have several advantages of other cell types, for example they can be generated in abundance from patients’ somatic cells (autologous) or those from a matched donor. iPSCs can be efficiently differentiated to functional endothelial cells (iPSC-ECs). Here, we used a murine excisional wound model to test the pro-angiogenic properties of iPSC-ECs in wound healing. Two full-thickness wounds were made on the dorsum of NOD-SCID mice and splinted. iPSC-ECs (5 × 10^5^) were topically applied to one wound, with the other serving as a control. Treatment with iPSC-ECs significantly increased wound perfusion and accelerated wound closure. Expression of endothelial cell (EC) surface marker, platelet endothelial cell adhesion molecule (PECAM-1) (CD31), and pro-angiogenic EC receptor, Tie1, mRNA was up-regulated in iPSC-EC treated wounds at 7 days post-wounding. Histological analysis of wound sections showed increased capillary density in iPSC-EC wounds at days 7 and 14 post-wounding, and increased collagen content at day 14. Anti-GFP fluorescence confirmed presence of iPSC-ECs in the wounds. Bioluminescent imaging (BLI) showed progressive decline of iPSC-ECs over time, suggesting that iPSC-ECs are acting primarily through short-term paracrine effects. These results highlight the pro-regenerative effects of iPSC-ECs and demonstrate that they are a promising potential therapy for intractable wounds.

## Introduction

Due to poor circulation and prolonged tissue ischaemia, many patients with peripheral arterial disease (PAD) develop chronic wounds or ulcers on their lower limbs, which can become infected and often necessitate amputation of the affected foot or leg. The complications associated with infection and amputation also lead to increased mortality in these patients, particularly in diabetics. Revascularization therapies improve wound healing in patients with critical limb ischaemia, yet chronic wounds remain a major individual and societal burden, with estimated global cost of care in excess of US$ 22 billion each year [1,2]. Current treatments for chronic wounds are largely focused on cleaning, infection control and debridement of dead tissue. The gold-standard in chronic wound care is the split-thickness autograft, which is a patch of skin taken from a healthy region and grafted on to the wound [3]. Other treatment options include bioactive dressings and donor keratinocytes, but these have limitations and additional therapies are required to better address the microvascular deficiency, which underlies the development of these wounds and contributes to their persistence and re-occurrence.

Acute wound healing comprises four overlapping stages; haemostasis, inflammation, proliferation and remodelling [4]. The inflammation and cellular proliferation phases take place from hours to several weeks after an injury and involve the recruitment of inflammatory cells to the wound site, formation of extracellular matrix (ECM) proteins and granulation tissue, keratinocyte migration, contraction and wound closure. Angiogenesis, the growth of new blood vessel networks, is a vital component of these processes and is dependent upon pro-angiogenic growth factors released by supporting cells, proliferation and migration of local endothelial cells into the wound bed, and recruitment of bone marrow-derived stem cells/endothelial progenitor cells (EPCs) [4–7]. The pathophysiology of chronic wounds is complex and disordered, but can be largely attributed to persistent inflammation and a lack of adequate tissue perfusion in the region, exacerbated by failure of ischaemia-mediated angiogenesis in the wound. Collagen deposition is also reduced in diabetic/PAD patient wounds and the collagen that is present is often glycated, which impairs the adherence of new keratinocytes to the ECM [8]. Inflammation is also known to be the primary driving force behind excessive scar formation when a wound does eventually heal [9]. Meanwhile, the inability of EPCs and supporting cells to form new blood vessel networks to clear necrotic debris and deliver oxygen and nutrients to the granulation tissue maintains the wound’s inflammatory status. Therapies that can effectively resolve either or both pathological processes are key to breaking the vicious cycle of failed healing in chronic wounds.

Stem cell therapy is a promising strategy to enhance angiogenesis and promote healing of chronic wounds. Adult stem and progenitor cells have been studied extensively in animal models of wound healing and appear to exert their beneficial effects via multiple mechanisms, including recruitment of other cells types, such as keratinocytes, macrophages and EPCs [10–12]. They have also been shown to produce collagen types I and III, which are critical for regeneration of the ECM and are the source of structural integrity and tensile strength in the healing tissue [13]. Clinical trials with bone marrow-derived stem cells are underway and there are a handful of published studies in both acute (surgical) and chronic (venous insufficiency and diabetic) wounds, which have reported consistent decreases in wound size, particularly with repeat treatments [14–17]. EPCs and embryonic stem cell derived endothelial cells have also been tested in animal models and improve wound healing via increased vascularization [18,19]. Indeed, a common beneficial feature of all stem cell therapies seems to be their pro-angiogenic effect.

Induced pluripotent stem cells (iPSCs) are derived by reprogramming somatic cells, such as dermal fibroblasts. They have unlimited self-renewal capacity and are therefore an abundant potential source of autologous or donor matched cells for therapy and may be more clinically translatable than other stem cell types. iPSCs can be differentiated to functional endothelial cells (iPSC-ECs) with high efficiency and reproducibility. We have shown previously that iPSC-ECs enhance angiogenesis and improve perfusion recovery in a mouse model of peripheral arterial disease [20,21]. The objective of the current study was to determine whether the pro-angiogenic properties of iPSC-ECs can be harnessed to promote wound healing. We demonstrate for the first time that iPSC-ECs enhance wound angiogenesis and perfusion, promote physiological collagen deposition and accelerate wound closure in a murine excisional wound healing model. These findings suggest that iPSC-ECs have potential as a treatment for chronic wounds and support further development of clinical grade iPSC-ECs for therapeutic angiogenesis.

## Methods

### iPSC reprogramming and differentiation to iPSC-ECs

Human iPSCs were generated via retroviral overexpression of Oct4, Klf4, Sox2, and c-Myc (OKSM) transcription factors in human dermal fibroblasts derived from healthy subjects. The iPSCs were differentiated to endothelial cells using previously described methods [20–23] (Supplementary Figure S1). The cells were transduced with a double fusion reporter construct encoding GFP and firefly luciferase, to enable *in vivo* fluorescence and bioluminescent imaging (BLI).

### Wound healing procedure

The wounding procedure was adapted from that previously described by Galiano et al. [24] and Dunn et al. [25]. Male NOD/SCID mice were used at 8–10 weeks of age. The operative region of the mouse’s back was prepared by removing the fur with clippers and a light depilatory cream, and two wound outlines were made, using a sterilized 5-mm biopsy punch. The skin in the middle of the outline was lifted using serrated forceps and full-thickness wounds were cut and excised using iris scissors. Silicone splints (approximately 10 mm diameter) were used to prevent wound closure via contraction. An adhesive was applied sparingly to one side of the splint and the splints were centred over the wounds. The splints were then secured in place using interrupted 6-0 PROLENE™ sutures (8805H, Ethicon LLC, San Lorenzo, Puerto Rico). After splinting, the mice were scanned with a laser Doppler (MOOR-LMD V192, Moor Instruments, U.K.) for wound perfusion measurement. They were placed on a heat mat in the prone position and the wound area was scanned three times per mouse per time point. Doppler scans were performed on alternate days post-wounding, up to and including day 14. Subsequent to the initial Doppler scan, cell treatments (5 × 10^5^ iPSC-ECs suspended in vehicle containing a 1:1 ratio of endothelial basal medium to growth factor reduced Matrigel) were injected into the wounds and the wounds were covered with adhesive Opsite™ dressings (66000041, Smith & Nephew, London, U.K.). All wound healing experiments and associated procedures were conducted in accordance with National Health and Medical Research Council (NHMRC) guidelines for the care and use of animals for scientific purposes and were approved by the Sydney Local Health District Animal Welfare Committee, Protocol #2014-004A.

### BLI

BLI was used for longitudinal tracking of iPSC-EC survival *in vivo* and was performed with an IVIS Lumina XRMS and Living Image software (version 4.5, PerkinElmer, Waltham, MA 02451, U.S.A.). The mice were anaesthetized with 2% isoflurane and d-luciferin (100 µl, 375 mg/kg) was administered by subcutaneous injection in a medial position immediately inferior to the wound sites. Bioluminescence intensity was calculated as the maximum mean radiance (photons/second/cm^2^/steradian) recorded in pre-defined ROI centred over the wounds. BLI was performed on alternate days post-wounding, up to and including day 14.

### Histological analysis of explanted wounds

Wound explants were fixed in 4% paraformaldehyde for up to 4 h at room temperature, then changed to 70% ethanol for at least 24 h. Paraffin infiltration was performed overnight by an automated tissue processor (Leica TP1020, Leica Biosystems Nussloch GmbH, Heidelberger Straße 17-19 69226 Nussloch, Germany). The infiltrated samples were then embedded in paraffin blocks for sectioning. Tissue samples were cut into 5-μm thick transverse sections using a rotary microtome, deparaffinized, and stained. Milligan’s Trichrome stain was performed to visualize collagen content. For immunohistochemistry analysis, paraffin sections were stained using immunohistochemistry techniques with primary antibodies anti-CD31 (Abcam, U.S.A.) for endothelial cells and anti-CD68 (Abcam, U.S.A.) for macrophages. Fluorescence microscopy was then used to visualize cell markers using Alexa Fluor 594 conjugated secondary antibodies. Anti-GFP staining was done using cryosectioning. Briefly, tissue was fixed in 4% paraformaldehyde for up to 4 h at room temperature then changed to 30% sucrose for at least 24 h. Tissue was then fixed in optimum cutting temperature (OCT) compound and kept at −80°C until cryosectioning. Samples were sectioned into 40-μm thick transverse sections and dropped into PBS in order for the residual O.C.T. to dissolve. Sections were then stained using standard free-floating techniques with a FITC-conjugated anti-GFP antibody (GeneTex). Both paraffin and cyrosections were then mounted and coverslipped with DAPI-containing mounting media (Vectashield, Vector Laboratories Inc., CA, U.S.A.).

### Analysis of host angiogenic gene expression in wounds

Total RNA was isolated from explanted control and iPSC-EC treated wounds with TRIzol reagent. Real-time quantitative PCR analysis was performed to evaluate the expression of murine vascular endothelial cadherin (VE-cadherin; sense: 5′-TCCTCTGCATCCTCACTATCACA-3′, antisense: 5′-GTAAGTGACCAACTGCTCGTGAAT-3′), vascular endothelial growth factor receptor-1 (Flt-1; sense: 5′-GAGGAGGATGAGGGTGTCTATAGGT-3′, antisense: 5′- GTGATCAGCTCCAGGTTTGACTT-3′), vascular endothelial growth factor receptor-2 (KDR; sense 5′-CCCTGCTGTGGTCTCACTAC-3′, antisense: 5′-CAAAGCATTGCCCATTCGAT-3′), platelet endothelial cell adhesion molecule (PECAM-1; sense: 5′-GAGCCCAATCACGTTTCAGTTT-3′, antisense: 5′-TCCTTCCTGCTTCTTGCTAGCT-3′), tyrosine kinase with Ig-like and EGF-like domains-1 (Tie-1; sense: 5′- CAAGGTCACACACACGGTGAA-3′, antisense: 5′-GCCAGTCTAGGGTATTGAAGTAGGA-3′), and vascular endothelial growth factor (VEGF; sense: 5′-TGCCAAGTGGTCCCAG-3′, antisense: 5′-GTGAGGTCTTGATCCG-3′). Glyceraldehyde-3-phosphate dehydrogenase (GAPDH; sense: 5′-GGGGCTCTCTGCTCCTCCCTGT-3′, antisense: 5′-CGGCCAAATCCGTTCACACCGA-3′) was used as a loading control. Relative gene expression was calculated as fold change compared with paired control wounds.

### Quantificative analysis of bioluminescence and histological images

Quantification of bioluminescence images was performed using LivingImage 4.5 (PerkinElmer) software. Histological and immunohistochemical sections were imaged using a Zeiss Upright Olympus fluorescence multichannel microscope, captured with a Nikon DP Controller 2.2 (Olympus, Japan). Immunohistochemical and histopathological analyses were done using ImageJ. Briefly, regions of interest (ROIs) were drawn around the wound site. For endothelial cells and fibroblasts, positive staining was quantified as individual particles counted based on a common threshold intensity. All cell types were quantified from *n*=6 sections per group per time point.

### Statistics

Data are expressed as mean ± S.E.M. and indicated in figures as *P<0.05, **P<0.01. The data were compared using paired Student’s *t* tests followed by Bonferoni’s post-hoc test using GraphPad Prism version 5.00 (GraphPad Software, San Diego, CA) for PC. IPSC-EC experimental samples were paired to control wounds within the same mouse.

## Results

### Engraftment of iPSC-ECs within wounds decreases following transplantation

**Figure 1:**
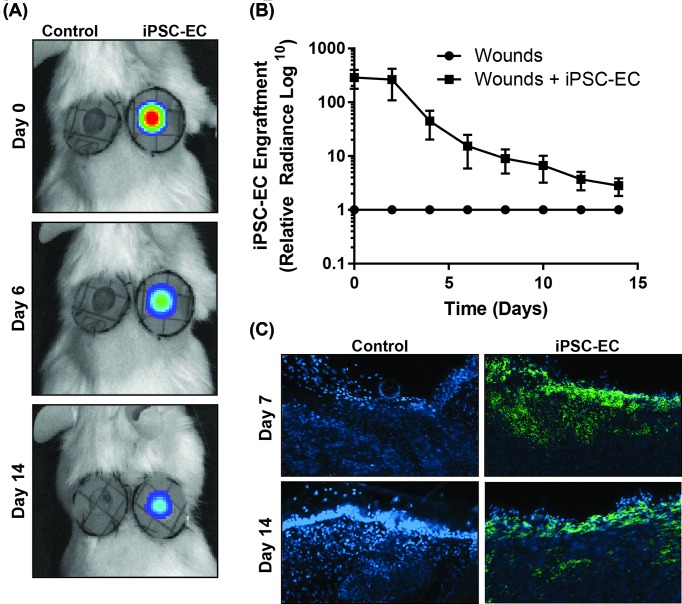
iPSC-EC engraftment in wounds iIPSC-ECs were transduced with a double fusion reporter construct encoding GFP for fluorescence imaging and firefly luciferase for BLI. (**A**) Representative IVIS images, showing bioluminescent signal present in iPSC-EC-treated wounds (right) on days 0, 6 and 14 post-wounding. (**B**) Declining bioluminescent signal in wounds over time, relative to control wound background signal. (**C**) GFP staining (green) with DAPI nuclear stain (blue) in control and iPSC-EC treated wounds at on days 7 and 14 post-wounding (*n*=6 per treatment group).

BLI was used to quantify the levels of iPSC-EC engraftment within wounds over time. Transfection of iPSC-ECs with a dual-report construct containing both the enhanced GFP (eGFP) and the firefly luciferase enzyme enabled visualization and quantification of transplanted iPSC-ECs. Intramuscular injection of d-luciferin substrate catalysed a bioluminescent reaction through the firefly luciferase enzyme within viable iPSC-ECs that could be visualized and quantified using the IVIS apparatus (Figure 1A). Bioluminescence quantification over 14 days showed that iPSC-EC presence in wounds decreases steadily over time (Day 0: 1.39 × 10^7^ ± 5.61 × 10^6^ compared with Day 14: 4.04 × 10^4^ ± 1.53 × 10^4^ photons/cm^2^/s/steradian). The largest decrease in bioluminescence occurred between days 2 and 4 (Day 2: 8.36 × 10^6^ ± 5.44 × 10^6^ compared with Day 4: 7.66 × 10^5^ ± 3.65 × 10^5^ photons/cm^2^/s/steradian, Figure 1B). Immunohistochemistry staining of the eGFP marker identified iPSC-ECs engrafted within the wound site at day 7, and a smaller proportion remaining at 14 days post-transplantation (Figure 1C).

### Engraftment of iPSC-ECs correlates with up-regulation of host angiogenic gene expression

**Figure 2:**
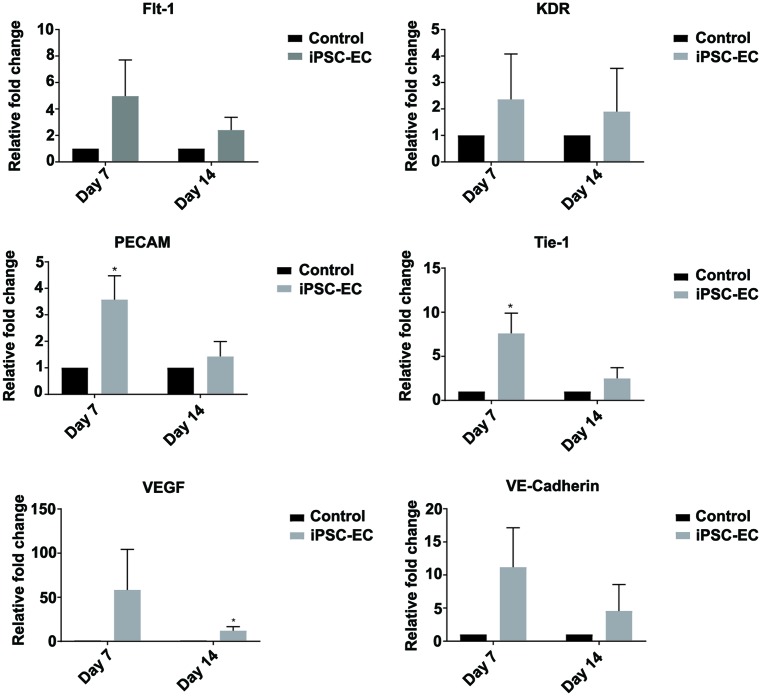
iPSC-EC treatment increases expression of pro-angiogenic genes qPCR analysis of wound mRNA revealed a significant up-regulation of PECAM (CD31) and endothelial cell receptor, Tie-1, expression in iPSC-EC wounds on day 7 post-wounding. This was resolved by day 14. No significant differences in endothelial cell surface marker VE-cadherin (CD144), VEGF, VEGF receptor 1 (Flt1) or VEGF receptor 2 (KDR) expression were measured (*P<0.05 compared with controls, *n*=6 per treatment group).

Functional angiogenesis is a complex process regulated by numerous cells types and growth factors. Ischaemic/injured tissues undergoing revascularization processes are often identified by the up-regulation of key angiogenic genes including VE-cadherin, VEGF, Flt-1, KDR, PECAM, and Tie-1. Wounds were explanted at days 7 and 14 for qPCR analysis to determine levels of these classical angiogenic genes. Increasing trends were observed in the expression of VE-cadherin, Flt-1, and KDR in iPSC-EC treated wounds when compared with control wounds at both time points, however no statistical significance was obtained. At day 7, *PECAM* and *Tie-1* genes were found to be significantly u-regulated by 350 ± 89 and 534 ± 134% in iPSC-EC treated wounds compared with control wounds (Figure 2). At day 14, VEGF expression was significantly up-regulated by 12 ± 4% in iPSC-EC treated wounds compared with controls (Figure 2).

### Wounds treated with iPSC-ECs exhibit increased angiogenesis

**Figure 3:**
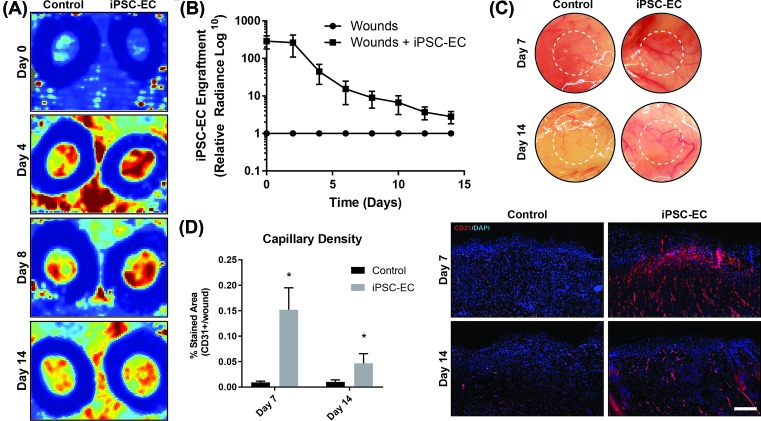
iPSC-EC treatment increases vascular density and wound perfusion (**A**) Representative laser Doppler images, showing perfusion in control and iPSC-EC treated wounds on days 0, 4, 8 and 18 post-wounding. (**B**) Wound perfusion in iPSC-EC treated wounds, relative to their respective control wounds. Increased perfusion in iPSC-EC treated wounds was most pronounced during the first week of healing. (**C**) Representative photomicrographs of wounds, showing increased neo-vessel formation in iPSC-EC wounds relative to controls. (**D**) Capillary density, as measured by CD31+ staining, was significantly increased in iPSC-EC wounds at both early and late time points (*P<0.05, *n*=6 per treatment group).

The anatomical hallmarks of angiogenesis include increased blood perfusion and high density of neo-capillary formation. Non-invasive IR laser Doppler imaging was conducted at the wound sites over 14 days to visualize and quantify levels of blood perfusion for the duration of wound healing (Figure 3A). Laser Doppler analysis revealed a maximum of two-fold increase in wound perfusion compared with control within the first 4 days following treatment in iPSC-EC treated wounds compared with control (Figure 3B). This gradually decreased back to baseline control wound perfusion levels by day 10. The subcutaneous layers of iPSC-EC treated, and control wounds were photographed following explant. A higher density of observable blood vessels was observed in iPSC-EC treated wounds compared with control (Figure 3C). To assess neo-capillary formation, cross-sections of wound explants were stained using the CD31 endothelial cell marker. At day 7, iPSC-EC treated wounds showed a 16-fold increase in capillary density compared with control wounds, which decreased to a four-fold increase by day 14 (Figure 3D).

### iPSC-EC treatment enhances the native wound healing process

**Figure 4:**
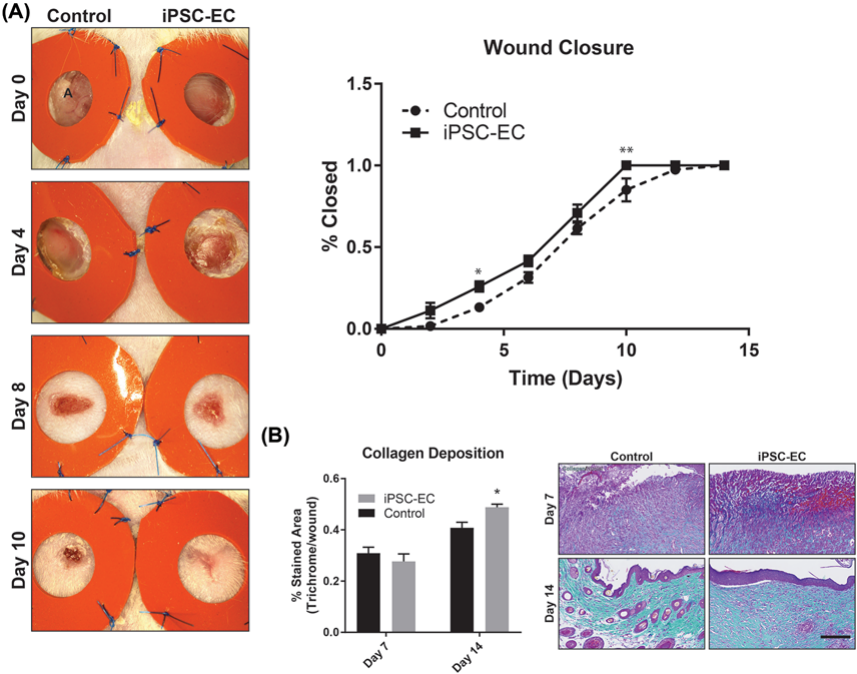
iPSC-EC treatment increases collagen deposition and accelerates wound closure (**A**) Representative photomicrographs, showing progressive wound closure over a 14-day period. Rate of closure was significantly increased in iPSC-EC treated wounds compared with controls. (**B**) Milligan’s Trichrome staining for collagen content. No differences were observed on day 7; by day 14 iPSC-EC treated wounds had significantly higher collagen content (*P<0.05, **P<0.01, *n*=6 per treatment group).

**Figure 5:**
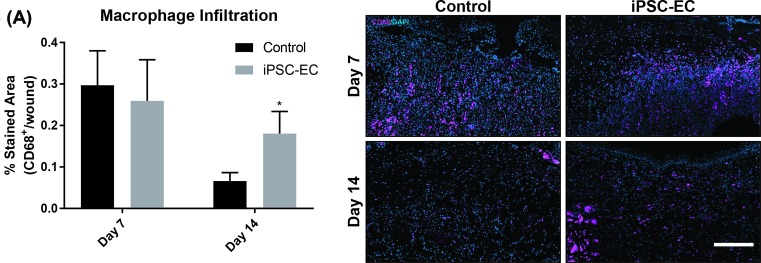
iPSC-EC treatment increases macrophage infiltration (**A**) Anti-CD68 staining for macrophage infiltration. No differences were observed on day 7; by day 14 iPSC-EC treated wounds had significantly macrophage infiltration (*P<0.05, *n*=6 per treatment group).

Native wound healing processes involve numerous cell phenotypes responsible for rebuilding the tissue architecture necessary for cell repopulation. Of these cell types, fibroblasts are largely implicated as essential matrix remodelling cells which facilitate neo-collagen deposition as a temporary matrix for cell growth and tissue regeneration. Using trichrome staining, wound cross-sections were quantified for levels of collagen deposition. iPSC-EC treated wounds showed a 20 ± 4% increase in collagen deposition at day 14 compared with control wounds (Figure 4B). Similar trends were observed with macrophage infiltration. At day 14, iPSC-EC treated wounds showed a 174 ± 6% increase in macrophage numbers compared with control wounds (Figure 5). The functional outcomes of wound healing were measured by taking three diameter measurements of wounds over 14 days and represented as percentage of total wound closure. iPSC-EC treated wounds show accelerated wound closure from day 2, which was significant on days 4 and 10 post-wounding and achieved complete wound closure 4 days earlier than control wounds (Figure 4A).

## Discussion

Chronic wounds and ulcers persist in a state of pathological inflammation, characterized by increased proteolytic activity, increased production of reactive oxygen species (ROS), senescent or dysfunctional fibroblasts, neutrophils and macrophages, and reduced pro-angiogenic growth factors and cytokines [4,7]. The aetiology of chronic wound formation in diabetes and peripheral arterial disease is multifactorial, but a major cause is underlying vasculopathy and diminished angiogenesis. The angiogenic response to ischaemia declines naturally with age and is further impaired in patients with vascular disease, who have reduced number and functionality of circulating EPCs [26–30]. The importance of angiogenesis in wound healing has been highlighted by interventional studies, which have shown that topical application of pro-angiogenic factors, such as VEGF or bFGF, is beneficial in accelerating diabetic wound healing in mice, while neutralizing them has the opposite effect [31–33]. However, clinical trials using single growth factors to boost angiogenesis have been largely unsuccessful, which likely reflects the complex regulation of angiogenesis *in vivo* and the need for a multifactorial approach [34].

Bone marrow and adipose tissue derived MSCs and EPCs have been in development for therapeutic angiogenesis for the past two decades, and have shown promise in promoting wound healing, however limited availability of healthy adult stem cells, and invasive aspiration procedures have tempered their use. iPSCs and their derivatives have several advantages, such as their relatively abundant supply, their non-controversial origins and the lack of immunogenicity if used for autologous transplantation [35]. Furthermore, the development of iPSC banking, the creation of multiple iPSC lines representing commonly present HLA allele combinations, means that patients could receive non-autologous cells without evoking an immune rejection, increasing the throughput while simultaneously reducing the time, cost and invasiveness of pluripotent cell therapies [36].

Here, we demonstrate the pro-angiogenic and wound healing capabilities of iPSC-ECs in a murine excisional wound model. Angiogenesis is vital for the formation and maintenance of granulation tissue in the first 2–4 days post-wounding. Neo-vessel formation typically begins around this time, peaking around day 7 before giving way to remodelling and maturation of the newly formed vasculature. We observed significantly accelerated wound closure in iPSC-EC treated wounds, as well as increased wound perfusion in the first week of healing, which was associated with a significant increase in endothelial cell surface marker PECAM-1 (CD31) staining in the wounds at both 7 and 14 days post-wounding. Consistent with previously described temporal patterns of angiogenesis in wound healing, absolute laser Doppler perfusion measurements decreased in both groups during the second week, approaching those of the surrounding healthy skin, as new dermis progressively covered exposed capillaries at the wound surface. Expression of *PECAM-1* and *Tie-1* mRNA was significantly up-regulated in iPSC-EC wounds on day 7 but was similar to expression in control wounds by day 14. Tie 1 is an endothelial cell specific orphan receptor, which has been shown to promote sprouting angiogenesis via regulation of Tie 2 receptor signalling [37].

iPSC-EC treatment also increased wound collagen deposition; sections from wounds treated with iPSC-ECs had significantly higher collagen content than the control wounds on day 14. Collagen deposition is vital for healthy wound healing, but the implication of increased collagen depends on the stage of healing as well as the types of collagen present; in the proliferative stage of healing collagen provides a matrix to support inflammatory and vascular cells as well as forming a stronger barrier than the fibrin clot [38]. In the late stages (remodelling), a reduction in collagen content is desirable to limit scarring. Collagen deposition begins within the first 24 h after wounding and peaks between 1 and 3 weeks later, depending on the wound site and size. As these wounds were still actively healing and in the proliferative phase at day 14, the observation of increased collagen is most likely indicative of a more advanced stage of healing compared with the controls, rather than excessive scarring. We also observed increased macrophage infiltration in iPSC-EC treated wounds at day 14 post-wounding, as evidenced by significantly increased CD68+ staining. In normal wound healing, macrophage infiltration peaks during the inflammatory phase (day 1–3 post-wounding) before subsiding. We observed no difference in CD68+ staining between control and iPSC-EC treated wounds at 7 days post-wounding. However, by day 14 iPSC-EC treated wounds had significantly greater CD68+ staining compared with their respective controls, suggesting sustained macrophage activity. This observation may be due to increased recruitment of macrophages in response to iPSC-ECs.

Poor cell survival and engraftment remains a major limitation to the long-term efficacy of cell therapies and therefore their suitability for clinical use. Most cells are lost within the first 48 h after administration, due to poor retention and cell death due to excessive inflammation, ischaemia or anoikis, the programmed death that occurs in endothelial cells and other anchorage-dependent cell types when they lose their interaction with ECM [39]. Very few previous wound healing studies included longitudinal tracking of the implanted cells to determine their eventual fate and those that did reported low rates of engraftment [40,41]. We used a firefly luciferase reporter gene construct and BLI for longitudinal tracking of iPSC-EC survival *in vivo* and although iPSC-EC fluorescent and bioluminescent signal was still detectable in the wounds 14 days post-treatment, we observed a substantial and progressive decline over the 2-week period. These data are consistent with previous *in vivo* survival of iPSC-ECs and suggest that the pro-angiogenic and pro-healing effects of our cells were primarily mediated via secretion of paracrine factors rather than engraftment or proliferation of the cells themselves [42]. Despite low rates of engraftment, stem cell treatments consistently evoke a beneficial response. This suggests that even small increases in retention could translate to sizeable improvements in tissue regeneration and long-term recovery and we are conducting further studies seeding iPSC-ECs on biomaterial scaffolds to determine if this can increase their *in vivo* longevity and potentiate their beneficial effects.

## Limitations

NOD/SCID mice are widely used in human haematopoietic cell studies and are an ideal strain for human cell transplantation because they lack functional B and T lymphocytes, lack the ability to mount an antibody-mediated response and additional deficiencies in their innate immune system allow for increased donor cell engraftment [43]. However, the blunted immune response to the cells creates a somewhat artificial environment in comparison with the situation in an immune competent organism. That being said, it is not possible to conduct pre-clinical, mechanistic investigations using human cells in other species without modulating the immune response. It is anticipated that the use of autologous or donor matched cells clinically would also mitigate the effects of immune rejection of the engrafted cells by the recipient, therefore we considered the NOD/SCID mouse to be the most suitable and appropriate model for these studies. It should also be noted that these mice are neither aged nor diabetic, so most innate wound healing capabilities are intact. This means that treatment effects are more difficult to detect, thus we anticipate an even more pronounced improvement after iPSC-EC treatment if we were able to use these cells in such a model.

Finally, we acknowledge that the iPSC-ECs used in the present study were differentiated from iPSCs that were generated using integrating viral vectors. These cells may not be suitable for use in a clinical setting because of the possibility of foreign DNA integrating into the host genome. However, the development of reprogramming protocols using small molecules means that iPSC-ECs for clinical applications can be generated without the use of viral vectors.

## Conclusion

In the present study, we have demonstrated for the first time that human iPSC-ECs have pro-angiogenic functionality in wound healing, promote fibroblast infiltration and collagen deposition and accelerate wound closure. Longitudinal tracking of iPSC-ECs *in vivo* revealed a progressive decline in surviving iPSC-ECs over time, indicating that the cells are acting primarily through short-term paracrine effects. These findings have provoked further studies optimising iPSC-EC delivery to improve *in vivo* survival and engraftment rates, and also make a strong case for the development of clinical grade iPSCs and their derivatives for therapeutic angiogenesis in patients with vascular diseases.

## Perspectives

- Ischaemia-mediated angiogenesis is impaired in patients with cardiovascular disease and diabetes, which leads to the development of intractable wounds and ulcers.
- Here, we show that human iPSC-derived endothelial cells (iPSC-ECs) promote angiogenesis, increase collagen deposition and accelerate healing in a murine wound healing model.
- Further development of human iPSC-ECs for therapeutic angiogenesis is a promising strategy to reduce the burden of chronic wounds in patients with peripheral arterial disease and diabetes.

## Supporting information

**supplementary Figure 1:** Generation of induced pluripotent stem (iPSCs) and induced pluripotent stem cell derived endothelial cells (iPSC-ECs) from dermal fibroblasts was achieved by retroviral overexpression of Oct4, Sox2, Klf4 and cMyc, followed by culture in endothelial lineage specific growth factors.

## Abbreviations

bFGF: basic fibroblast growth factor
BLI: bioluminescent imaging
ECM: extracellular matrix
EGF: epidermal growth factor
eGFP: enhanced GFP
EPC: endothelial progenitor cell
iPSC: induced pluripotent stem cell
iPSC-EC: iPSC-derived endothelial cell
MSC: mesenchymal stem cell
NOD-SCID: non-obese diabetic - severe combined immunodeficiency
OCT: optimal cutting temperature
PAD: peripheral arterial disease
PECAM-1: platelet endothelial cell adhesion molecule
ROI: regions of interest
Tie-1: tyrosine kinase with Ig-like and EGF-like domains-1
VEGF: vascular endothelial growth factor
VE-cadherin: vascular endothelial cadherin
