# Supplementary material for: Induced pluripotent stem cell-derived endothelial cells promote angiogenesis and accelerate wound closure in a murine excisional wound healing model
Source: Biosci Rep. 2018 Jul 31;38(4):BSR20180563. doi: 10.1042/BSR20180563 (PMC6066657; doi:10.1042/BSR20180563)
Supplement: Supplementary file 1 [file bsr20180563_Supp1.pdf]

Supplementary figure 1. Generation of induced pluripotent stem (iPSCs) and induced pluripotent stem cell derived endothelial cells (iPSC-ECs) from dermal fibroblasts was achieved by retroviral overexpression of Oct4, Sox2, Klf4 and cMyc, followed by culture in endothelial lineage specific growth factors.

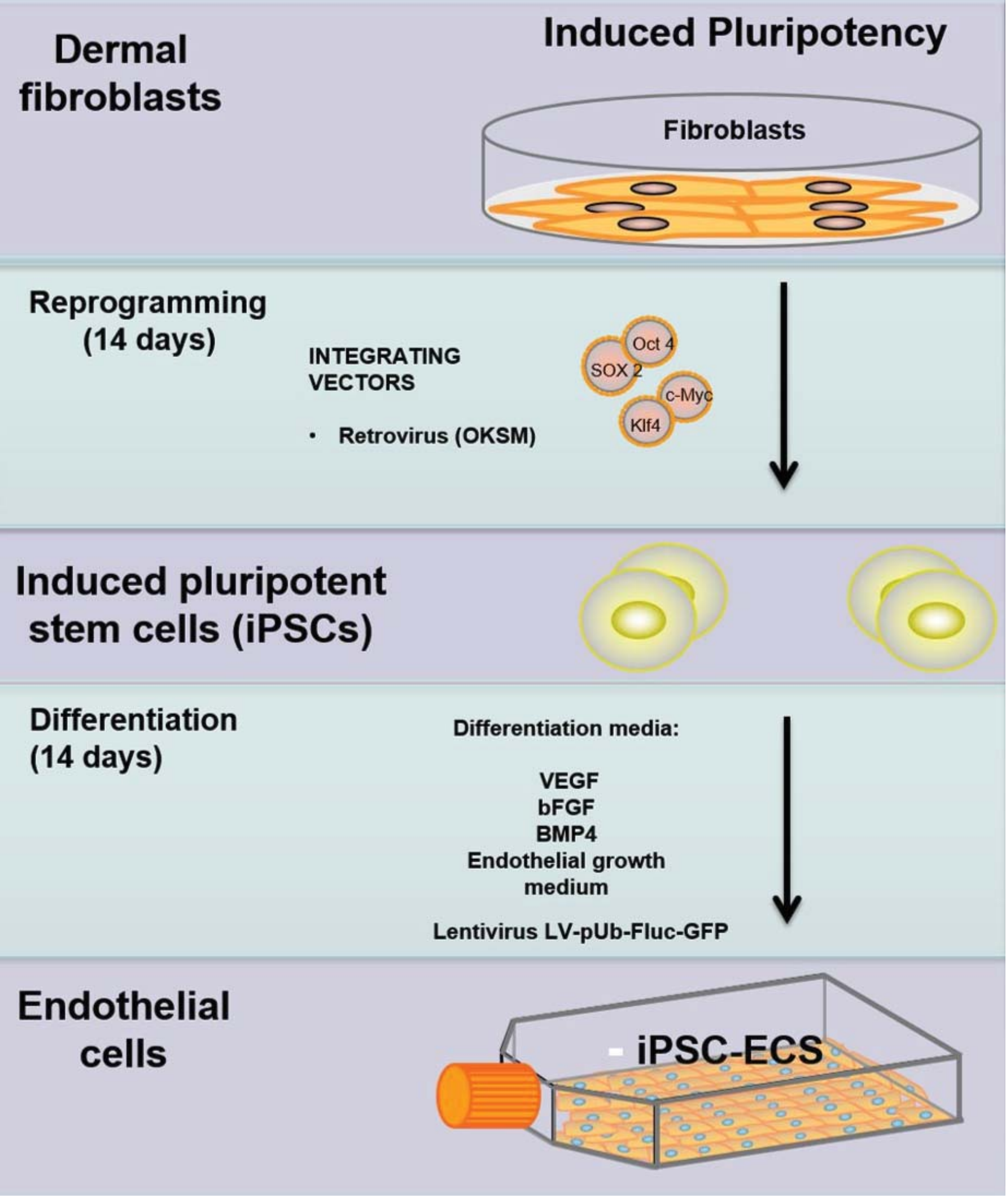

Dermal fibroblasts
Induced Pluripotency
Fibroblasts
Reprogramming (14 days)
INTEGRATING VECTORS
• Retrovirus (OKSM)
Oct 4
SOX 2
c-Myc
Klf4
Induced pluripotent stem cells (iPSCs)
Differentiation (14 days)
Differentiation media:
VEGF
bFGF
BMP4
Endothelial growth medium
Lentivirus LV-pUb-Fluc-GFP
Endothelial cells
iPSC-ECS
